# Speaking to the past

**DOI:** 10.1038/s41597-020-0531-6

**Published:** 2020-06-30

**Authors:** Harry Dowsett

**Affiliations:** Florence Bascom Geoscience Center, U.S. Geological Survey, Reston, 20192 Virginia USA

**Keywords:** Climate change, Research data, Palaeoclimate

## Abstract

“Speak to the past and it shall teach thee”. I first read those words on a dedication tablet within the John Carter Brown library at Brown University where I was a graduate student. Little did I know the phrase would accurately describe the next three and a half decades of my career. Paleoclimate data are the language we use to look into the past to understand ourselves and ultimately our future.

Our changing climate is an existential threat to the environment, infrastructure, and public health, often dominating political, economic and cultural dialogues. The latest climate models project conditions for the end of this century that are generally outside of the human experience^1,2^. Instrumental data extends the climate record back in time by perhaps a couple of centuries and historical records, e.g. written records of storms, harvest yields, and phenological changes, several thousand years for some regions. Deep-time records of paleoclimate provide insight into the climate system over millions of years sampling conditions very different from the present day, and in some cases similar to model projections for the future. Thus, paleoclimatology provides essential context for the scientific understanding of climate change needed to inform international policy decisions.

Paleoclimatology does not just provide isolated, anecdotal facts about the past. By integrating paleoclimate data gleaned from geological archives with computer modelling, we learn important lessons about how the climate system may respond under conditions markedly different than present day^3^. Past intervals of both recent and deep-time provide estimates of climate sensitivity to greenhouse gas forcing, magnitude and rates of change, as well as impacts of change on the biosphere, hydrosphere and cryosphere. Paleoclimate data are the foundation of how we understand the inner workings of the climate system and its behavior under different conditions, and thereby inform adaptation strategies related to the ecological health of the environment.

Paleoclimate data encompass an array of data types and methodologies^4^. An informal three-part organization might be primary, secondary, and model-generated data (Fig. 1). **Primary** data are observations, collections and measurements. Examples include population censuses or quantitative counts of fossil taxa, tree rings, measurements of stable isotopes or trace elements incorporated in preserved fossil material or other natural archives, or measurements of biomarkers contained in sediments. **Secondary** data are derived by analysis and calibration of present-day primary data to climate variables like sea-surface temperature or mean annual rainfall, providing a means to produce estimates of climate variables (e.g. temperature, salinity, precipitation) for times in the past. It is important to note that while secondary data (interpretations of primary data) may change with new understanding, primary data never change and thus retain value far beyond the purpose for which they were developed. Time series of secondary data from a single site can provide information about the temporal evolution of climate at that location. Secondary data from a specific chronologic horizon at a number of locations provides a snapshot of regional or even global paleoclimate conditions. The combination of time-series and time-slice data allow a better understanding of the dynamic development and evolution of climates and environments. Paleoclimate data can also be derived from reconstructions based upon model simulations, model-produced bioclimatic variables, and re-analysis products using data assimilation techniques. These **model-generated** data sets provide high spatial and temporal resolution reconstructions that facilitate research on the causes of climate variability and the impacts of climate change on environmental, ecological, and evolutionary studies.

**Fig. 1:**
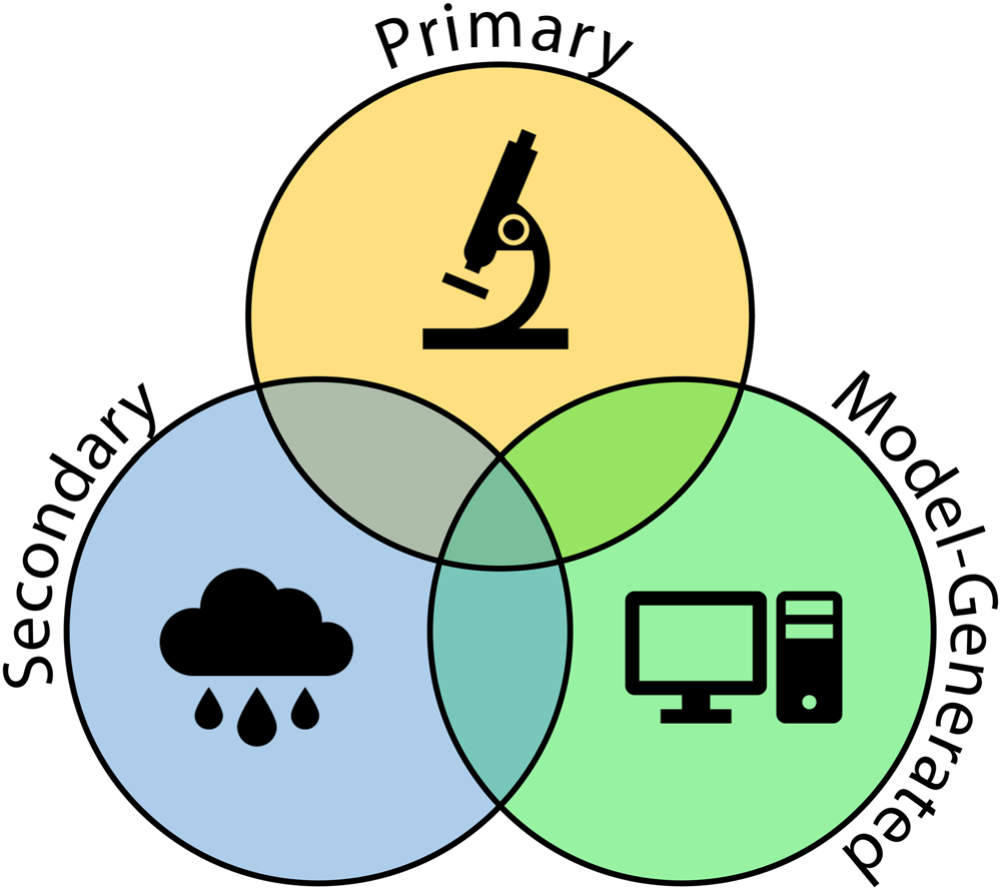
Paleoclimate data classification. Primary data include observations, measurements and collections. Secondary data are climate variables derived by interpretation of primary data. Model-generated data include simulations, model-produced bioclimatic variables, and re-analysis products generated or assimilated using climate models.

Paleoclimate model output can be compared to secondary paleoclimate data to better evaluate and understand results^5–8^. Areas of agreement and disagreement in these data – model comparisons inform us, and through an iterative process, often lead to improvements in both our understanding the paleoclimate data and confidence in models.

## Paleoclimate data descriptors included in this collection

The data descriptors included in this collection touch on many aspects of paleoclimate including calibration, chronologic framework, primary data, secondary data, time series, computer-generated climate variables, and regional and global spatial reconstructions. They provide detailed information about data sets that goes beyond meta-data.

This collection is being launched concurrently with a new reconstruction analysis of the Holocene paleoclimate published by Kaufman *et al*.^9^, derived from a rigorously compiled set of proxy data already published at the journal by these and other authors who contributed data and helped make them reusable^10^. The database of paleotemperature records by Kaufman *et al*.^10^ assembles a carefully vetted collection of globally distributed terrestrial and marine time series within a high-resolution chronologic framework, covering the past 12,000 years. This data product is an extremely valuable resource with many applications for understanding environmental change over the Holocene Epoch. For example, Kaufman *et al*.^9^ analyze the Holocene database using a variety of statistical methodologies to derive a robust record of global mean surface temperature of the Earth over the past 12,000 years.

We hope other researchers will share palaeoclimate data in the spirit exemplified by the publications in this evolving collection, and we invite our readers to use these data – these messages from the past – to help us better understand ourselves and our shared future.
